# Association of vitamin D receptor gene polymorphisms with metabolic syndrome: a case–control design of population-based cross-sectional study in North China

**DOI:** 10.1186/1476-511X-13-129

**Published:** 2014-08-09

**Authors:** Yi Zhao, Sha Liao, Jun He, Yanan Jin, Hailong Fu, Xiaoying Chen, Xuemin Fan, Hongxia Xu, Xiuying Liu, Jing Jin, Yuhong Zhang

**Affiliations:** School of Public Health, Ningxia Medical University, 1160 Shengli Street, Yinchuan, Ningxia P.R. China; Centers for Disease Control and Prevention in Ningxia, 180 Baohu Road, Yinchuan, Ningxia P.R. China; Department of Clinic Administrations, Ningxia People’s Hospital, 301 Zhengyuan Street, Jingfeng District, Yinchuan, 750004 Ningxia PR of China; Affiliated Hospital of Ningxia Medical University, 804 Shengli Street, Yinchuan, Ningxia P.R. China

**Keywords:** Vitamin D Receptor gene, Polymorphisms, Metabolic syndrome

## Abstract

**Background:**

Metabolic syndrome (MS) increases a risk of developing cardiovascular disease (CVD) and type 2 diabetes mellitus (T2DM). The Vitamin D Receptor gene (VDR) may be important for developing MS. The aim of this study is to investigate the correlation between the VDR gene polymorphisms and MS in North China.

**Methods:**

A case–control study included 391 participants with MS according to the MS diagnostic criteria of International Diabetes Federation 2005 (IDF2005) and 400 controls was conducted on the basis of a cross sectional study which was performed from 2008 to 2012 in Ningxia Hui Autonomous Region, China. Anthropometric data, blood pressure and blood samples were collected in the field investigation. Blood biochemistry analyses were carried out in the laboratory. Two single-nucleotide polymorphisms (SNPs) in the VDR gene, BsmI (rs1544410 A > G) and FokI (rs 2228570 C > T), were genotyped.

**Results:**

The difference in the occurrence of genotypes in BsmI between individuals with MS and the control group was significant. Compared with genotype Bb/bb and allele b, genotype BB and allele B showed higher frequencies in MS cases than controls, which suggested they were risk factors. In addition, the genotype BB carriers with MS presented a higher waist circumference, while genotype FF for the FokI polymorphism was correlated with lower BMI in subjects with MS.

**Conclusion:**

Our study suggests that the VDR gene polymorphisms appear to be associated with MS in the Northern Chinese population. Allele B and BB genotype for BsmI are risk factors for MS. The BsmI polymorphism seems to influence waist circumference, while the FokI polymorphism influence BMI in subjects with MS.

## Background

Metabolic syndrome (MS) includes a variety of metabolic components in abnormal pathological state, which contains abnormal plasma glucose, dyslipidemia, high blood pressure, and high cholesterol [1]. Patients with MS have a greater risk of developing cardiovascular disease (CVD) and type 2 diabetes mellitus (T2DM) [2–4]. For the past few years, the prevalence of MS has been on the rise in adults globally, increasing from about 20% to 40% [5–8] in multiple groups of people, and is becoming a serious global health burden. Although insulin resistance [9] and central obesity [10] are considered to be its primary etiological factors, genetics undoubtedly plays an important role as well. In particular, genetic influence may be different among various ethnic groups [11].

The Vitamin D receptor (VDR) is a member of the steroid hormone receptor family that acts as a transcriptional activator of many genes. The DNA polymorphisms that have been often reported for the VDR gene are (described in both restriction sites and dbSNP info): BsmI (rs1544410 A > G), FokI (rs 2228570 C > T), TaqI (rs731236 T > C), and ApaI (rs 7975232 C > T). In past years, many studies have found that these polymorphisms are related to bone mineral density(BMD) [12], calcium metabolism [13], tuberculosis, hepatitis [14] and cancer [15, 16]. Additionally, some of these polymorphisms have been recently identified to be associated with type 1 diabetes [17], T2DM [18], and insulin secretion [19].

Although the molecular mechanisms of VDR in components of MS remain unclear, a relationship between 25(OH)D_3_ and the disorders of MS has been previously demonstrated [20–23]. Studies have also discussed VDR gene polymorphisms for associations with the components of MS, which suggest that two major VDR gene polymorphisms (BsmI and FokI) seemed to influence BMI, insulin resistance, and serum HDL cholesterol [24, 25]. However, little is known about the role of VDR gene polymorphisms in MS for Chinese population.

Therefore, we suppose the two single-nucleotide polymorphisms (SNPs), BsmI (rs1544410 A > G) and FokI (rs 2228570 C > T), of the VDR gene intricately affect the development of MS. This study was designed to determine the contribution of VDR polymorphisms to MS in a population from North China.

## Materials and methods

### Participants

A cross-sectional study was conducted from 2008 to 2012 in Guyuan County, Qingtongxia County and Pingluo County, of Ningxia Hui Autonomous Region, China. Stratified cluster sampling was applied to select two villages (one Hui ethnic village and one Han ethnic village) in each county, totaling six villages. During the study period, 4614 subjects aged 24–75 were interviewed with a questionnaire, followed by anthropometric measurements (height, weight, waist circumference), and blood pressure determination. Pregnant or breastfeeding women, patients with severe mental disease and other serious illness were excluded from the study. The subjects enrolled did not report to have chronic viral infection, cold or flu, acute respiratory infection, or any type of surgery in the week preceding the study. Meanwhile, 2615 participants had blood drawn via venipuncture for laboratory measurements, and finally 1169 blood samples were selected for genotyping by mechanical sampling (k = 1). Based on diagnostic criteria (International Diabetes Federation IDF-2005), 391 cases with MS and 400 controls were selected from the same population to conduct the case–control study. The study protocol had been approved by the Medical Ethics Review Committee of Ningxia Medical University. All participants signed the consents on enrollment after they received written and verbal information about the study.

All participants were interviewed by trained persons with a standard closed-ended questionnaire. The contents of the questionnaire included general demographic characteristics, blood pressure, blood lipid, blood sugar, smoking, physical activity status, drinking and related diseases (including hypertension, coronary heart disease, stroke and diabetes medical history and family history, individual treatment, etc.).

### Anthropometric measurements and blood pressure

Standing height was measured using a portable ruler. Body weight was also measured using a weight scale (Omron, China). All measurements were performed by well-trained investigators. The reading of measurements was accurate within 0.1 cm or 0.1 kg, and the average of the two collected measures was recorded for further analysis. Body mass index (BMI) was calculated as kg/m^2^.Obesity was defined as BMI ≥ 30. Arterial blood pressure was measured three times in sitting position using an electronic sphygmomanometer (Omron-HEM 7301-IT, China). All participants were at rest for at least 30 minutes before the measurement was collected. Patients with average blood pressures ≥140/90 mmHg or taking antihypertensive medication in 2 weeks were classified as being hypertensive.

### Blood biochemistry analyses

Blood samples were drawn from the antecubital vein at the hours of 6 to 8 AM, after at least 10 hours of fasting and avoidance of alcohol. Two sets of fasting blood samples were collected separately from each subject in sodium fluoride potassium oxalate tubes (for glucose) and lithium heparin vacuum tubes (for lipids).The latter collections were then centrifuged and kept at −80°C until analysis. Fasting glucose was immediately determined by One Touch Ultra 2 (LifeScan, USA). Serum levels of HDL- cholesterol, LDL- cholesterol, and triglycerides were measured by enzymatic assay (CHOD-PAP, Roche Diagnostics GmbH). All analyses were carried out through an automatic biochemical analyzer (COBASE 501, Roche Diagnostics GmbH). Subjects were further divided into two groups, cases with MS and controls without MS, according to the IDF definition. For a subject to be defined as having MS, they must have: Central obesity (waist circumference ≥ 90 cm in males, ≥ 80 cm in females, or BMI is >30 kg/m^2^) along with any two of the following four factors: triglycerides ≥ 150 mg/dL (1.7 mmol/L); HDL-cholesterol < 40 mg/dl (1.03 mmol/L) in males, < 50 mg/dL(1.29 mmol/L) in females; blood pressure, systolic BP ≥ 130 or diastolic BP ≥ 85 mmHg; fasting glucose ≥5.6 mmol/L (100 mg/dl) [1].

### Genotyping

Genomic DNA was extracted from the whole blood with the SE Blood DNA Kit (OMEGA, USA). After extraction, 30 samples of the extracted material were randomly selected for validation by agarose gel electrophoresis. SNP genotyping of BsmI and FokI in the VDR gene was performed using a technology known as SNPscan™ [26–28](Genesky Biotechnologies, China). SNPscan™ is a patent technology and developed on double ligation and multiplex fluorescence PCR with high genotyping accuracy (>99.9%) and call rate (>98%). Specifically, highly multiple ligation products in different length were generated on account of high specificity of the ligase reaction. Meanwhile, the ligation probes and templates were further lengthened through double ligation. With blue fluorescent dye modified universal primers, ligation products were amplified by Thermal Cycler (ABI, USA) and then detected through fluorescent capillary electrophoresis. Finally, GeneMapper (Life Technologies) software was used to read the data.

### Statistical analysis

Statistical analysis was conducted using SPSS statistical software (version 14.0 SPSS Corp, College Station, TX) and descriptive data were presented by mean and standard deviation. The Kolmogorov-Smirnov test was used to analyze normality of the distribution of each variable. Comparisons between any two groups were performed using independent t-tests. Chi-square analysis was applied to examine the variation of sex, genotype and gene frequency in different MS groups. Chi-square analysis was also used to test Hardy–Weinberg equilibrium (HWE) for the genotypes in all groups of subjects. ANOVA analysis was used to analyze clinical variables in different genotypes. Odds ratios (ORs) and their 95% confidence interval (CI) were computed for the risk alleles from logistic regression analysis. Statistical significance was set at P < 0.05.

## Results

### General characteristics and components of MS

Characteristics of the subjects with and without MS are shown in Table 1. In our study, the mean age of the 391 participants with MS was 53 (±11) years, and the mean age of 400 controls was 53 (±11) years. Compared with the controls, BMI, waist circumference, blood pressure, fasting glucose, and triglycerides were significantly higher in individuals with MS, while HDL cholesterol was lower. Both VDR BsmI and FokI genotypic distributions in controls were in Hardy-Weinberg equilibrium (*P*, 0.762 and 0.376, respectively) (Table 2).

**Table 1:** **Population Characteristics and components of MS**

	MS	Control	***P***-value
Male/Female, n	99/292	100/300	0.917
Age	53.37 ± 10.99	53.73 ± 11.08	0.997
Height(cm)	159.09 ± 8.11	157.73 ± 7.34	0.018
Weight(kg)	66.19 ± 10.15	56.09 ± 7.90	<0.001
BMI	26.09 ± 2.97	22.53 ± 2.72	<0.001
WC(cm)	89.70 ± 6.63	79.11 ± 7.47	<0.001
BPS(mmHg)	136.88 ± 20.38	124.87 ± 18.29	<0.001
BPD(mmHg)	84.13 ± 11.55	77.19 ± 11.01	<0.001
FBG(mmol/L)	6.15 ± 1.31	5.58 ± 0.83	<0.001
TG(mmol/L)	2.07 ± 1.19	1.30 ± 1.16	<0.001
HDL-C(mmol/L)	1.28 ± 0.38	1.48 ± 0.34	<0.001

WC, Waist circumference; BPS, Systolic blood pressure; BPD, Diastolic blood pressure; FBG, fasting blood-glucose; TG, triglycerides; HDL-C, HDL- cholesterol.

**Table 2:** **Hardy-Weinberg equilibrium of control group**

Genotype		Predictive value	Observed value	χ ^2^	***P***-value
BsmI	CC	328.52	328	0.092	0.762
	CT	67.97	69		
	TT	3.52	3		
FokI	AA	84.39	80	0.783	0.376
	AG	198.22	207		
	GG	116.39	112		

### The distribution of VDR BsmI and FokI polymorphisms

As shown in Table 3, both genotypic and allelic frequencies of VDR BsmI and FokI polymorphisms were presented for participants with or without MS. Genotypes of BsmI and FokI are normally expressed as dominant homozygous genotype “BB, FF”, heterozygous genotype “Bb, Ff”, and recessive homozygous genotype “bb, ff”. There were differences in genotypes and gene frequencies of VDR BsmI between the MS cases and controls, with P-values of 0.011 and 0.03 respectively. Frequency of genotype BB and Bb was significantly different between individuals with MS and without MS, after comparing all of the three genotypes with each other (**χ**^**2**^ **=** 7.022, P = 0.008). However, there was no difference in FokI polymorphism.

**Table 3:** **Genotypes and gene frequencies**

	BsmI					FokI				
	BB	Bb	bb	B	b	FF	Ff	ff	F	f
**MS, n**	347	42	1	736	44	75	184	132	334	448
**Control, n**	328	69	3	725	75	80	207	112	367	431
**χ** ^**2**^	--			9.016		3.073			1.720	
***P*** **-value**	0.011*			0.03		0.215			0.19	

*Fisher's Exact Test; BB/FF normally show dominant homozygous genotype; Bb/Ff show heterozygous genotype; bb/ff show recessive homozygous genotype.

### VDR polymorphisms and MS risk

The difference in the occurrence of the genotypes in BsmI between individuals with MS and the control group was significant (*P*trend = 0.02). The frequencies of genotype BB and combined Bb/bb in individuals with MS were 89% and 11% respectively, in the control group were 82% and 18% respectively, which suggested that the genotype BB was a risk factor (*P* = 0.006; OR = 1.771; 95% CI, 1.179-2.661). Compared with genotype BB, genotype Bb was likely to be a protective factor (*P* = 0.009; OR = 0.575; 95% CI, 0.381-0.869). The incidences of B and b alleles for the BsmI polymorphism in the two group were statistically significant (allele B vs. b; *P* = 0.005), which indicated allele b could be a protective factor. However, it seemed no significant evidence to be risk factors or protective factors to FokI polymorphism in individuals with MS (Table 4).

**Table 4:** **FokI and BsmI polymorphisms and MS risk**

Genotype	MS n(%)	Control n(%)	***P***-value	OR	95% CI
BsmI^a^					
BB(CC)	347(89.0)	328(82.0)		1	
Bb(CT)	42(10.7)	69(17.3)	0.009	0.575	0.381-0.869
bb(TT)	1(0.3)	3(0.7)	0.318	0.315	0.033-3.044
Bb + bb	43	72		1	
BB	347	328	0.006	1.771	1.179-2.661
B	736(94.4)	725(90.6)		1	
b	44(5.6)	75(9.4)	0.005	0.578	0.393-0.850
FokI^b^					
FF(AA)	75(19.2)	80(20.0)		1	
Ff(AG)	184(47.1)	207(58.9)	0.779	0.948	0.653-1.376
ff(GG)	132(33.7)	112(28.1)	0.266	1.257	0.840-1.882
Ff + ff	316	319		1	
FF	75	80	0.759	0.946	0.666-1.345
F	334(42.7)	367(46.0)		1	
f	448(57.3)	431(54.0)	0.190	1.142	0.936-1.393
BBFF^c^	69	68		1	
BBFf	164	174	0.761	0.929	0.624-1.382
BBff	114	85	0.211	1.322	0.854-2.046
BbFF	5	11	0.156	0.448	0.148-1.358
BbFf	19	32	0.111	0.585	0.303-1.131
Bbff	18	26	0.276	0.682	0.343-1.358
bbFF	0	1	1.000	0.000	0.000
bbFf	1	1	0.992	0.986	0.060-16.077
Bbff	0	1	1.000	0.000	0.000

^a^ *P*trend = 0.02; ^b^ *P*trend = 0.216; ^c^ *P*trend = 0.187;BB/FF normally show dominant homozygous genotype; Bb/Ff show heterozygous genotype; bb/ff show recessive homozygous genotype.

Moreover, the genotypes of BsmI and FokI were combined and generated as 9 new genotypes like BBFF to analyze the cumulative effect of the polymorphisms, as shown in Table 4. But there were no significant differences in the risk of MS (*P*trend = 0.187).

### The associations of VDR gene polymorphisms with the components of MS

Table 5 presented the distribution of some clinical variables according to the genotypes observed in individuals with MS and without MS. Because of the limited frequencies of genotype bb in the VDR BsmI polymorphism, we combined Bb and bb genotype as a group. For the VDR BsmI variant, individuals with MS carrying BB genotype presented a higher waist circumference than individuals with Bb + bb genotype, whilst genotype BB was associated with higher waist circumference and BMI in individuals without MS than seen in others. Additionally, the presence of FF genotype for the FokI polymorphism was associated with lower BMI than that with Ff/ff genotype in individuals with MS. In addition, to the individuals without MS, higher systolic blood pressure was seen in subjects with Ff genotype than that with ff genotype, and subjects with genotype FF/Ff presented a higher diastolic blood pressure than individuals with ff genotype. Moreover, the values of the remaining variables were very similar among all subjects for both VDR BsmI and FokI polymorphisms.

**Table 5:** **FokI and BsmI polymorphisms and MS components**

		***BsmI***(rs1544410 A > G)		***P***	***FokI***(rs 2228570 C > T)			***P***
		BB	Bb + bb		FF	Ff	ff	
**MS**	BMI	26.1 ± 3.1	25.7 ± 2.2	0.396	25.1 ± 2.5^a^	26.3 ± 3.1	26.4 ± 2.9	0.005
WC(cm)		90 ± 6.6	87.6 ± 6.3	0.025	88.7 ± 5.5	89.7 ± 7	90 ± 6.6	0.284
BPS(mmHg)		136.4 ± 19.8	141.9 ± 22.7	0.096	135.9 ± 20	137.5 ± 20.6	136.6 ± 20.4	0.829
BPD(mmHg)		84.1 ± 11.4	85 ± 12.5	0.625	84 ± 11.8	84.8 ± 11.3	83.2 ± 11.8	0.438
FBG(mmol/L)		6.1 ± 1.2	6.4 ± 2.2	0.163	6.2 ± 1.6	6.1 ± 1	6.2 ± 1.5	0.496
TG(mmol/L)		2.1 ± 1.2	2 ± 1.2	0.788	2.1 ± 0.9	2.1 ± 1.3	2 ± 1.2	0.821
HDL-C(mmol/L)		1.3 ± 0.4	1.4 ± 0.3	0.177	1.2 ± 0.3	1.3 ± 0.3	1.3 ± 0.5	0.077
**Control**	BMI	22.7 ± 2.8	22 ± 2.4	0.030	22.5 ± 2.9	22.5 ± 2.6	22.5 ± 2.8	0.995
WC(cm)		79.7 ± 7.6	76.3 ± 6.1	0.000	78.7 ± 8	79.2 ± 6.5	79.2 ± 8.7	0.849
BPS(mmHg)		124.9 ± 18.3	124.9 ± 18.3	0.985	124.9 ± 18.6	126.8 ± 19.3^b^	121.5 ± 15.6	0.045
BPD(mmHg)		77.2 ± 11.1	77.1 ± 10.8	0.939	78.2 ± 10.5	78.1 ± 11.8	74.9 ± 9.4^a^	0.031
FBG(mmol/L)		5.6 ± 0.9	5.6 ± 0.5	0.727	5.5 ± 0.8	5.7 ± 1	5.5 ± 0.5	0.118
TG(mmol/L)		1.3 ± 1.2	1.2 ± 0.6	0.254	1.3 ± 0.6	1.3 ± 1.5	1.2 ± 0.6	0.789
HDL-C(mmol/L)		1.5 ± 0.3	1.5 ± 0.3	0.227	1.4 ± 0.3	1.5 ± 0.3	1.5 ± 0.3	0.596

^a^significant for the genotype Ff/ff; ^b^significant for the genotype ff.

## Discussion

The results showed that VDR polymorphisms may influence the emergence of MS. The VDR BsmI (rs1544410 A > G) polymorphism appeared to associate with MS. Specifically, allele B and genotype of BB in BsmI were associated with MS, while allele b and genotype Bb seemed to play a protective role. The role of genotype bb was unclear, because of its limited frequency. When discussed VDR gene polymorphisms with the components of MS, we found the genotype BB carriers with MS presented a higher waist circumference. In addition, genotype FF for the VDR FokI (rs2228570 C > T) polymorphism was correlated with lower BMI in MS. Our findings were different with previous researches which demonstrated the VDR FokI polymorphism was related with triglycerides and HDL-cholesterol levels [25].

A few studies have demonstrated that VDR polymorphisms were related to obesity, diabetes, insulin sensitivity and insulin secretion [17, 19, 29]. VDR BsmI and FokI polymorphisms have been previously reported to be associated with anthropometric and biochemical parameters describing MS. Trzmiel et al. (2008) found that VDR BsmI polymorphism seemed to influence BMI, while the FokI VDR polymorphism appeared to affect insulin sensitivity and serum HDL cholesterol in men [24]. However, Lwow et al. (2008) indicated that VDR BsmI polymorphism did not seem to predispose postmenopausal women to obesity and insulin resistance, but the genotype BB was connected with dyslipidemia [30]. Frey et al. (2003) did not find evidence for the association of VDR polymorphisms with glycemia either [31]. Results of these study seemed to conflict with the pleiotropic effect of the VDR gene in individuals with MS, and this result was not found in other populations [32]. Moreover, interactions in circulating glucose, triglyceride, cholesterol and insulin levels were not well explained [33, 34]. For this reason, whether these differences come from ethnic variations or interactions in components of MS still needs further exploration, and we should not focus on the disorders in MS separately.

The VDR gene is not a main influencing factor to the variability of circulating levels of vitamin D, according to genome-wide association (GWA) studies [17, 35, 36]. However, it is clear that the VDR gene plays a primary role in the pleiotropic actions of 1,25(OH)_2_D_3_ [37] and in insulin secretion [19, 38]. Serum 25-hydroxyvitamin D levels is inversely related to percentage body fat content in healthy women [20] and incident hypertension [21]. Furthermore, McKeown et al. (2011) found that the predicted 25-hydroxyvitamin D score might be an important determinant for change in fasting plasma glucose concentration in the Framingham offspring study [22]. Unfortunately, our study did not include serum 25-hydroxyvitamin D, but we could assume that serum 25-hydroxyvitamin D is the focus leading metabolic disorders if the interactions we discussed above were taken into consideration.

## Conclusion

This study revealed a significant association of VDR BsmI polymorphism with MS in the Northern Chinese population. Allele B and genotype BB for BsmI are risk factors for MS. The BsmI polymorphism seemed to influence waist circumference, while the FokI polymorphism influence BMI in subjects with MS. Thus, we propose that the BsmI and FokI polymorphisms of VDR are potential prognostic variables which may predict the risk of developing MS. Even so, further examination should be carried out on large population. Also, genome wide association studies are still needed to evaluate the direct effect of these polymorphisms on MS.

## Authors’ information

Yi Zhao and Sha Liao Co-first authors.

## Abbreviations

MS: Metabolic syndrome
VDR: Vitamin D Receptor gene
CVD: Cardiovascular disease
T2DM: Type 2 diabetes mellitus
IDF2005: International Diabetes Federation 2005
SNPs: Single-nucleotide polymorphisms
BMD: Bone mineral density
BMI: Body mass index
WC: Waist circumference
BPS: Systolic blood pressure
BPD: Diastolic blood pressure
FBG: Fasting blood-glucose
TG: Triglycerides
HWE: Hardy–Weinberg equilibrium
CI: Confidence interval
GWA: Genome-wide association.
